# Bioactivity and metabolic profiling of crude extracts from endophytic bacteria linked to *Solanum mauritianum* scope: Discovery of antibacterial and anticancer properties

**DOI:** 10.1016/j.heliyon.2024.e40525

**Published:** 2024-11-20

**Authors:** Abraham Goodness Ogofure, Ezekiel Green

**Affiliations:** a Department of Biotechnology and Food-Technology, Faculty of Science, University of Johannesburg, P. O. Box 17011, Doornfontein, Johannesburg, 2028, South Africa

**Keywords:** Bacterial endophytes, Secondary metabolites, Cytotoxic activity, *Pantoea* species, *Arthrobacter* sp., *Bacillus licheniformis*

## Abstract

Bacterial endophytes associated with *Solanum mauritianum* Scop. represent a promising source of novel bioactive compounds with potential antibacterial and anticancer properties. This study aimed to investigate the diversity, distribution, and bioactivity of crude extracts derived from endophytic bacteria, focusing on their effects against bacterial pathogens of public health relevance and two cancer cell lines. Fresh, healthy plant samples were collected, and endophytes were isolated using standard cultural techniques. Identification of the endophytes was carried out through conventional and molecular methods. The comprehensive profiling and characterization of crude secondary metabolites were conducted using Liquid Chromatography-Quadrupole Time-of-Flight Mass Spectrometry (LC-QTOF-MS/MS) and Gas Chromatography-High Resolution Time-of-Flight Mass Spectrometry (GC-HRTOF-MS). The antibacterial activity and minimum inhibitory concentration were evaluated for the secondary metabolites using the Resazurin Microtitre assay. The anticancer activity of the metabolites was evaluated against A549 Lung carcinoma cells and U87MG Glioblastoma cells (ATCC culture cell lines). The result revealed a diversity of bacterial endophytes, including *Pantoea* species, *Luteibacter* sp. *Bacillus safensis, Arthrobacter* sp., and *Bacillus licheniformis.* These endophytes displayed distinct-tissue-specific distribution patterns within *S. mauritianum*. Metabolic profiling of three endophytes (*P. ananatis*, *B. licheniformis,* and *Arthrobacter* sp.) revealed 14 common and numerous unique metabolites. The crude secondary metabolites exhibited broad-spectrum antibacterial activity against reference strains of *Bacillus cereus*, *Pseudomonas aeruginosa*, and *Staphylococcus epidermidis*, where MICs as low as 0.125 mg/ml were recorded across several secondary metabolites of *Pantoea ananatis*, *Bacillus licheniformis*, and *Arthrobacter* sp. The cytotoxicity assays on UMG87 glioblastoma and A549 lung carcinoma cells revealed that the secondary metabolites did not induce cell death but instead promoted cell proliferation with different viability rates. While this proliferative effect limits their direct application as anticancer agents, it raises intriguing possibilities for their role in tissue regeneration or repair. This study provides critical insights into the microbial diversity of *S. mauritianum* and underscores the potential of its endophytic bacteria as sources of bioactive compounds with diverse biotechnological applications.

## Highlights

• Novel bioactive compounds with potential antibacterial properties were identified from bacterial endophytes.
• Crude extracts from three bacterial strains showed strong antibacterial activity with MIC as low as 0.125 mg/mL.
• Metabolites showed no anticancer activity but indicated possible tissue-regenerative effects on A549 and UMG87cells.
• Tissue-specific bacterial endophytes from *Solanum mauritianum*, reveal insight into endophyte-plant interactions.
• Study underscores bacterial endophytes as a sustainable source of bioactive compounds for durg development.

## 1 Introduction

Bacterial endophytes, residing asymptomatically within plant tissues, have garnered significant attention due to their potential to produce a diverse array of bioactive secondary metabolites [[1], [2], [3]]. These secondary metabolites often exhibit remarkable pharmacological properties, including antimicrobial and anticancer activities, thus presenting a promising avenue for drug discovery and development [1,2,4,5]. The intricate relationship between plants and their endophytic microbiota has emerged as a frontier in natural product discovery and biotechnological applications. *Solanum mauritianum* Scop., commonly known as woolly nightshade, is a perennial shrub native to South America that has naturalized in various parts of the world, including South Africa [[6], [7], [8]]. While the plant has been primarily considered an invasive species in many regions, it has a history of ethnomedicinal use, suggesting the presence of bioactive compounds [9,10]. However, the endophytic community of this plant and its potential as a reservoir of novel bioactive metabolites remain largely unexplored, as very little information is known about the endophytic communities in *S. mauritianum* as well as the secondary metabolites produced by its microbiome and their potential for use in medicine and pharmacology. In recent decades, the advancement in high-resolution analytical techniques, particularly in liquid chromatography coupled with mass spectrometry (LC-MS) and gas chromatography-mass spectrometry (GC-MS), has revolutionized the field of metabolomics [[11], [12], [13], [14]]. These powerful tools enable comprehensive profiling of complex mixtures of secondary metabolites produced by endophytic bacteria, facilitating the identification of novel compounds [15] and elucidation of their chemical structures. The global health challenges posed by antimicrobial resistance and the persistent need for innovative cancer therapies [[16], [17], [18]] underscore the importance of exploring new sources of bioactive compounds using metabolomic approaches with even further prospects for elucidating the mechanisms of action of these metabolites. Bacterial endophytes, with their unique ecological niche and evolutionary adaptations, represent an untapped resource for the discovery of novel molecules (secondary metabolites) with potential pharmaceutical and medical applications for the overall benefit of global health. This study aims to provide a comprehensive analysis of the secondary metabolite profiles of bacterial endophytes isolated from different plant parts of *S. mauritianum* Scop. We employ state-of-the-art analytical techniques to characterize the chemical diversity of these endophytes and evaluate their antibacterial and anticancer activities against cell lines. By investigating the bioactive potential of these endophytic bacteria, we seek to uncover novel lead compounds for drug development and gain insights into the chemical ecology of plant-endophyte interactions. Our findings may not only contribute to the discovery of new therapeutic agents but also enhance our understanding of the complex relationships within the plant microbiome and their implications for natural product biosynthesis.

## 2 Methodology

### 2.1 Collection of sample and preparation

Healthy and fresh parts of *S. mauritianum* samples (fruits, stem and leaves) were collected at the DFC of the University of Johannesburg. A Botanist authenticated the plant, and it was immediately transported to the laboratory for analysis, where it was washed thoroughly with sterilized distilled water. The surface sterilization of the plant parts using absolute ethanol was performed after rinsing with sterile distilled water. Subsequently, a weighed portion (10g) of the samples was treated with Tween 20 at a concentration of 5 % for 5 min, with vigorous vortexing. The samples were then thoroughly rinsed multiple times with sterile distilled water to eliminate any remaining traces of Tween 20 [19].

The samples were sterilized for 1 min in 70 % ethanol and then rinsed five times with sterile distilled water to ensure complete removal of the ethanol. Furthermore, the samples were sterilized for 10 min using a 1 % bleach solution (sodium hypochlorite) to guarantee complete elimination of any epiphytic microorganism. The plant samples which were sterilized using the bleach solution were rinsed 10 times with sterile distilled water, and 100 μL of the last rinse water was used as a negative control (by aseptically plating in triplicate on nutrient agar) to validate the surface sterilization process [20].

### 2.2 Isolation of bacterial endophytes

Aside from plating the last rinse water on nutrient agar, the samples (after rinsing) had their exterior surfaces carefully removed before they were macerated in PBS (phosphate buffer saline). The macerated samples were then serially diluted using a 10-fold dilution factor as described by Ogofure and Igbinosa [21], and 100 μL of all the serially diluted tubes, beginning with 10^1^ to 10^3^, were plated in triplicates on nutrient agar using spread plate technique. The plates were incubated for 5 d, at a temperature of 30^0^ C, with periodic examination of the plates from the first to fifth day for the presence of microbial growth. The control culture plates (100 μL of the last rinse water) were also evaluated for the presence of bacteria or fungi. For any negative control plate with microbial growth (indicating an ineffective sterilization process and the presence of epiphytes), the associated plant sample culture was discarded. The entire procedure was then repeated until the complete elimination of epiphytes was confirmed. Sample culture plates with growth (specifically without growth for control) were sub-cultured to purify the bacterial isolates further. Preservation of the isolates was carried out with 50 % glycerol (1:1 glycerol and bacterial broth culture) and thereafter stored at −80^0^ C.

### 2.3 Conventional and molecular identification of bacterial isolates

The bacterial isolates were identified and characterized using conventional and molecular techniques. Gram-stained reaction and cell morphology were evaluated using standard methods delineated by Collins et al. [22]. The cell morphology and Gram reaction were confirmed after the Gram staining technique with the aid of an Olympus CH20BIMF200 (Ina, Japan) compound bright-field microscope at a magnification of 1000x.

The molecular assay began with the culture of pure bacterial colonies at 30^0^ C in nutrient broth for 24 h. The overnight broth cultures were centrifuged at 13000×*g*, with the supernatant of the centrifuged broth discarded after it was decanted [23]. The extraction of genomic bacterial DNA was carried out using a Zymo research bacterial DNA kit (catalogue number R2014, Irvine, USA). The manufacturer's specification was strictly adhered to for the extraction process and the extracted DNA was quantified using a NanoDrop UV–VIS (ThermoFisher Scientific ND-2000, USA). Forward and reverse primers (16S-1492R:5′-CGGTTACCTTGTTACGACTT-3′ and 16S-27F: 5′-AGAGTTTGATCMTGGCTCAG-3′) were used for the amplification of the 16S rRNA gene to specifically target bacteria DNA at 1500 base pairs. The polymerase chain reaction (PCR) process was carried out using a standardized buffer and a PCR master mix (2x). The cleaning of the PCR products was done using ExoSAP-it™ strictly following the instructions of the manufacturer. The sequencing of the gene was carried out in Inqaba Biotech (Pretoria, South Africa), and the phylogenetic analysis was conducted using PhyloT and iTOL (Interactive Tree of Life).

### 2.4 Extraction of secondary metabolites from bacterial endophytes

A quadruplicate determination of a 5-L Schott bottle containing nutrient broth (3 L) was used for the culture of bacteria from different plant parts of *S. mauritianum* according to methods delineated by Hagaggi and Mohamed [24]. The broth cultures were agitated at 200×*g* (relative centrifugal force), equivalent to 1276 RPM, for a duration of 7 d. After cultivation, 60 g/L of sterilized Amberlite® XAD7HP resin (20–60 mesh, Sigma-Aldrich, Cat. No. BCBR6696V) was used to adsorb organic compounds from the culture. The resin-culture mixture was incubated at room temperature and centrifuged at 200×*g* for 2 h. The resin was subsequently separated from the samples via filtration using a sterilized cheesecloth. The cheesecloth was thoroughly washed with acetone, and the acetone-soluble fraction was further concentrated using a rotatory evaporator. In a sterilized measuring cylinder(s), the extracts from the different isolates were transferred in an equivalent ratio (1:1 v/v) of ethyl acetate. The resulting mixtures were agitated vigorously for 10 min, and into sterile separating funnels, they were decanted and allowed to separate gently. The entire process was repeated until the production of a viscous dark brown colour liquid, which would turn light-yellow following the removal of acetone. Fractions of the ethyl acetate were removed by rotatory evaporation, and the extracts were preserved on a cool and dry shelf inside amber-coloured bottles for further use. The light-yellow liquid obtained was evaporated to ensure the collection of every bit of the extract.

### 2.5 Antibacterial activity and minimum inhibitory concentration (MIC) of crude secondary metabolites

The crude extracts of the secondary metabolites derived from the endophytes were weighed at 0.176 g and then placed into sterile McCartney vials. Serial dilutions were made from 32 mg/mL to 0.0625 mg/mL concentrations from a stock concentration of 64 mg/mL in 0.1 % DMSO (dimethyl sulfoxide). The outermost wells of the plates were filled with sterile distilled water, and 100 μL of standardized bacterial culture corresponding to 1.5 x10^8^ cells/mL (0.5 McFarland's standard) of overnight broth cultures were added to the wells 2 to 11 horizontally, and the B to G vertically. The experiment was conducted using quintuplicate determination (five independent replicates) for each bacterial endophyte. In the vertical order, approximately 100 μL of each diluted sample was pipetted into the wells, starting from the highest concentration of 32 mg/mL and proceeding down to the lowest concentration of 0.0625 mg/mL. The plates were incubated at 37^0^ C for 18–24 h before the addition of 10 μL of Resazurin sodium salt solution (0.02 % w/v) to the wells, wrapped with foil and further incubated for 2 h. This experiment also included both positive and negative controls in Streptomycin (3.2 μg/mL) and sterile distilled water, respectively. A colour change from blue resazurin to pink is indicative of bacterial growth or metabolic activity. Therefore, the MIC was regarded as the lowest concentration where there were no visible or noticeable changes in the wells after the addition of resazurin when further incubated for 2 h [9,25].

### 2.6 Anti-cancer activity of crude secondary metabolites from endophytes

The crude extracts (secondary metabolites) from the bacterial endophytes were also evaluated for their anticancer activities using the method delineated by Sebola et al. [25], where a stock solution of crude extract was diluted from 100 μg/ml to 3.13 μg/ml. The bacterial secondary metabolites were evaluated for anticancer properties against A549 Lung carcinoma cells and U87MG Glioblastoma cells (ATCC culture cell lines). The positive control employed in this assay was auranofin (100–3.13 μg/mL), which has been reported by Li et al. [26] and also by Roder and Thomson [27] to have excellent activity against lung cancer cell lines. The percentage of cell viability was evaluated using the method stipulated by Handayani et al. [28] after employing the formula below:

$$\%\;cell\;viability=\frac{Ea-Ba}{Ca-Ba}\;x\;\left(100\right)$$

Where Ea = absorbance of the extract, Ba = absorbance of the blank, and Ca = absorbance of the control.

The National Cancer Institute (NCI) has established guidelines for interpreting the cytotoxic activity of crude extracts. According to these guidelines, a crude extract is considered to possess cytotoxic activity if its half-maximal inhibitory concentration (IC50) value is less than or equal to 20 μg/mL after either 48 or 72 h of incubation.

### 2.7 Comprehensive analysis of secondary metabolites in bioactive crude extract using Liquid Chromatography-Quadrupole Time-of-Flight Mass Spectrometry (LC-QTOF-MS/MS)

A comprehensive analysis of secondary metabolites in the bioactive crude extracts from three selected endophytic bacterial strains with the most activity was evaluated in this study. The LC-QTOF-MS/MS system was an ultra-high-performance liquid chromatography system produced by ThermoScientific (Erlangen, Germany) that was attached to a Compact™ QTOF, also produced in Germany (Bruker Daltonics) that uses an ESI (electrospray ionization) interface. The entire process involves the chromatographic separation of analytes in RP-UHPLC (Reverse Phase Ultra-High-Performance Liquid Chromatography) with a 5 μL injection volume. This was done through a Raptor ARC-18 column (a product of Restek, USA) with 100 mm length, 2.1 mm internal diameter, 2.7 μm particle size, and a 90 Å pore size as the dimension of the raptor. The control and operation of the system and as well data acquisition were carried out using the HyStar software version 2.10 (Bruker, Germany). The total analysis run time was 40 min. 0.1 % formic acid acetonitrile (Solvent B) and 0.1 % formic acid in water (Solvent A) were used as the mobile phases in the experiment. The gradient flow started at 5 % in 2.0 min, increased to 95 % in 28 min, stayed isocratic at 95 % for 5 min, and then decreased to 5 % in 1 min. Specific ESI (+) parameters were set for the total analysis run time, and within a range of 50–1300 *m*/*z*, the mass spectra were obtained in centroid mode [29,30].

### 2.8 Preparation of crude extracts (secondary metabolites) from bacterial endophytes for LC-QTOF-MS/MS

The preparation of samples for the comprehensive analysis of secondary metabolites began by adding 1 mg/mL (w/v) of crude endophyte extracts in methanol (HPLC grade, produced by Merck, SA). Sonication (for 8–10 min) of the crude extract was done until it fully dissolved. Filtration was carried out for the samples using 0.22 μm PVDF (polyvinylidene fluoride) syringe filters into 1 mL autosampler vials (LC-MS). The blank and control used for the HPLC experiment were methanol (HPLC grade) and the media (nutrient agar) used to grow the bacteria.

### 2.9 Gas Chromatography-High Resolution Time-of-Flight Mass Spectrometry (GC-HRTOF-MS) settings and analysis of secondary metabolites

The comprehensive analysis of secondary metabolites was conducted using a state-of-the-art GC-HRTOF-MS system (LECO Corporation) in conjunction with LC-QTOF-MS technology. For GC-HRTOF-MS analysis, crude extracts (1 mg) were dissolved in analytical-grade methanol, vortexed, and filtered through a 0.2 μm syringe filter before transfer to autosampler vials. The system employed a Pegasus instrument coupled with an Agilent 7890A chromatograph and a multipurpose autosampler, operating in high-resolution mode. Chromatographic separation was achieved using a Rxi®-5 ms column (30 m × 0.25 mm ID x 0.25 μm), with helium as the carrier gas at a flow rate of 1 mL/min. The injection volume was 1 μL in splitless mode, with transfer line and inlet temperatures set at 225 °C and 250 °C, respectively. The oven temperature program initiated at 70 °C (0.5 min hold), ramped to 150 °C at 10 °C/min (2 min hold), and finally to 330 °C at 10 °C/min (3 min hold). Electron ionization was performed at 70 eV, with an MS acquisition rate of 13 spectra/second over an *m*/*z* range of 30–700. Data processing utilized LECO Chroma TOF-HRT software, with metabolite identification based on Feihn Maillib and NIST libraries, considering compounds with similarity values exceeding 70 % [15].

### 2.10 Data analysis and visualization

The software employed for the data analysis in this study includes the Bruker Compass data analysis software (version 4.3) for spectral data interpretation. The MetFrag web tool (version 2.1) was used for the characterization of the fragment spectra connected to databases such as ChemSpider, PubChem, and KEGG. For some specific and accurate results, specific settings were applied in MetFrag.

Data visualization was performed using R software version 4.3.3 and various specialized packages [31]. Heatmaps were generated using the *pheatmap* package [32] to visualize metabolite profiles and cytotoxicity data, with custom colour scales and hierarchical clustering applied where appropriate. The *ggplot2* package [33] was employed for creating enhanced heatmaps and other complex visualizations, allowing for fine-tuned aesthetics and the incorporation of numerical data within cells. For comparative analysis of secondary metabolites across bacterial endophytes, a Venn diagram was constructed using the *VennDiagram* package [34], with customized positioning of labels and colour schemes to optimize clarity. Statistical analyses, including clustering and correlation assessments, were conducted using base R functions and supplementary packages as needed [30]. All visualizations were optimized for clarity and scientific presentation, with attention to colour choice, font sizes, and layout to ensure effective communication of the datasets.

## 3 Results and discussion

**Fig. 1:**
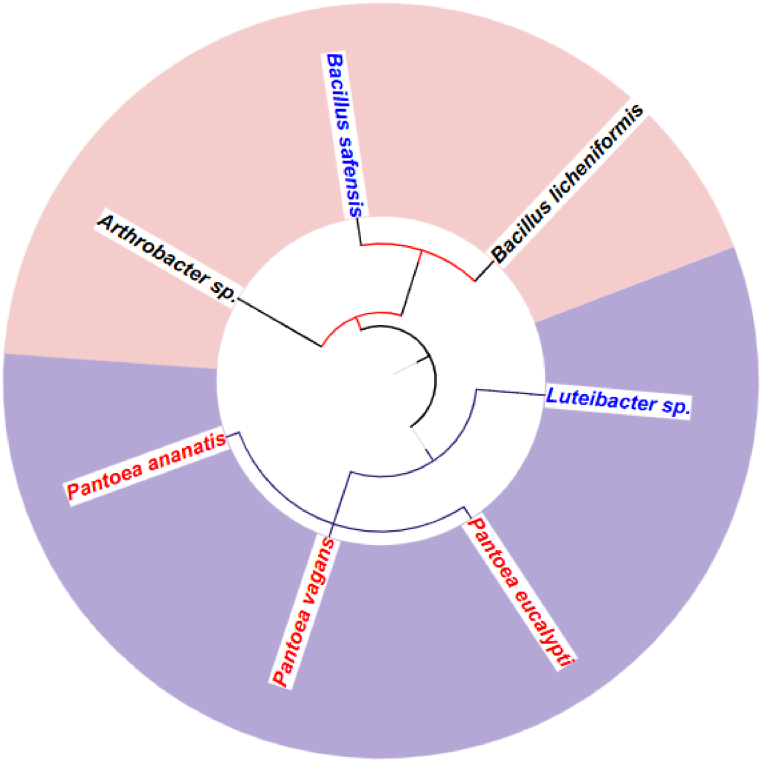
Identification and phylogenetic analysis of bacterial endophytes from *S. mauritianum.* The seven bacterial endophytes were deposited in the gene bank with the following accession numbers: MK070326, MK070327, MK070328, MK070329, MK070330, MK070331, and MK554845.

The phylogenetic analysis of the seven bacterial endophytes isolated from *S. mauritianum* Scop. provides insights into the diversity and tissue-specific distribution of these bacteria. The generated figure (Fig. 1), created using iTOL (Interactive Tree of Life) and PhyloT, highlights the presence of endophytes from three different bacterial classes: Firmicutes, Actinobacteria, and Gamma proteobacteria. Notably, red nodes represent bacteria from the class Firmicutes, black nodes indicate Actinobacteria (or Actinomycetes), and purple nodes correspond to Gamma proteobacteria. A notable pattern was observed in the tissue-specific distribution of endophytes. *Pantoea* species (red ink) were predominantly isolated from stem tissues, while *Luteibacter* and *Bacillus safensis* (blue ink) were primarily recovered from fruits. *Arthrobacter* and *Bacillus licheniformis* (black ink) were predominantly associated with leaf tissues. These findings highlight the influence of plant tissue type on the composition of endophytic communities and underscore the potential for discovering novel and tissue-specific microbial adaptations. This distribution pattern suggests that *S. mauritianum* harbours a diverse array of bacterial endophytes from different bacterial phyla. The findings furthermore highlight the influence of plant tissue type on the composition of endophytic communities and underscore the potential for discovering novel and tissue-specific microbial adaptations.

**Fig. 2:**
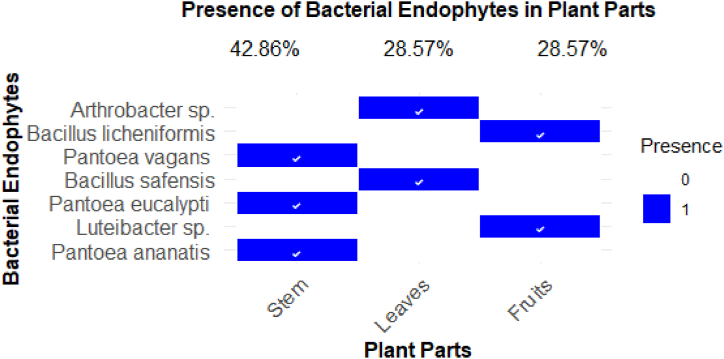
Heatmap illustrating the distribution of seven bacterial endophytes across different parts of *S. mauritianum*.

The heatmap (Fig. 2) presents a detailed distribution of seven bacterial endophyte species across different plant parts, including the stem, leaves, and fruits. Notably, the stem is the most colonized plant part, with three bacterial species present, accounting for 42.86 % of the total bacterial colonization. All identified *Pantoea* species (*Pantoea vagans*, *Pantoea eucalypti*, and *Pantoea ananatis*) were localized in the stem, suggesting a tissue-specific affinity for this genus, potentially due to the unique ecological or physiological conditions within the stem that favour the colonization of these endophytes in this plant tissue. In contrast, the leaves and fruits each hosted two different bacterial species, representing 28.57 % of the colonization for each part, none of which were from the *Pantoea* genus. This divergence in bacterial genera between plant parts highlights the variability in microbial colonization, likely influenced by distinct microenvironments within the plant tissues. The leaves were found to harbor *Arthrobacter* sp. and *Bacillus safensis*, while the fruits contained *Arthrobacter* sp. and *Bacillus licheniformis*. This distribution pattern underscores the specialization of certain bacterial genera to specific plant parts, reflecting complex plant-microbe interactions that could be crucial for understanding the plant's overall health and disease resistance. These insights into tissue-specific microbial communities can provide valuable information for future studies on plant-microbe relationships and their potential applications in agriculture and biotechnology.

The findings in this study were consistent with the report of Lin et al. [35], who reported the distribution pattern of endophytic bacteria in tea plants with stems and leaves having the most diverse endophytic bacterial communities for the above-ground parts of the plants asides from the root. The abundance of diverse *Pantoea* species in the stem tissue of *S. mauritianum* was found to correlate with the report of Gao et al. [36], who made similar findings from the stem of a maize plant. Endophytic bacteria with tissue-specific affinity have been reported to have some plant growth-promoting functions specific to the tissues in which they are found. For example, *Bacillus* species isolated from leaves in this study were also reported to enhance the growth of Strawberry plants by increasing the leaf number [37]. The tissue-specificity of endophytic bacteria was also affirmed by the report of Wu et al. [38], who reported that *Bacillus* is the most dominant genus exhibiting not only tissue specificity but also geographical specificity. Climate has also been reported to affect the diversity of endophytes in plants through its indirect effects on the land ecosystem [39].

Furthermore, the presence of Firmicutes, Actinobacteria and Gamma proteobacteria in the aerial parts of *S. mauritianum* Scop. was also consistent with the report of Andreolli et al. [40], who suggested that Actinobacteria and Firmicutes are frequently associated with plants above 3 years old, while Gamma and Alpha-proteobacteria are associated with older plants. This finding also suggests that the diversity and microbiota composition of endophytic bacteria in plants are time-dependent or time-bound, thereby providing some useful clues concerning the concept or phenomenon of age-related resistance (ontogenic resistance) in plants. The information is necessary because the survivability of any plant is hugely dependent on the plant's endophytic community and composition [41]. In this study, the endophytic communities and composition in the leaves and fruits were more diversecompared to the stem. Consistent with this finding are the reports of Chaudhry et al. [42] and Almario et al. [43], who opined that leaf-microbial interactions are crucial to the overall health and vitality of the plant with the leaf-microbial communities exhibiting reproducible patterns and dynamics in promoting the growth of the plant through the presence of certain core taxa like the Firmicutes (*Bacillus* species).

**Fig. 3:**
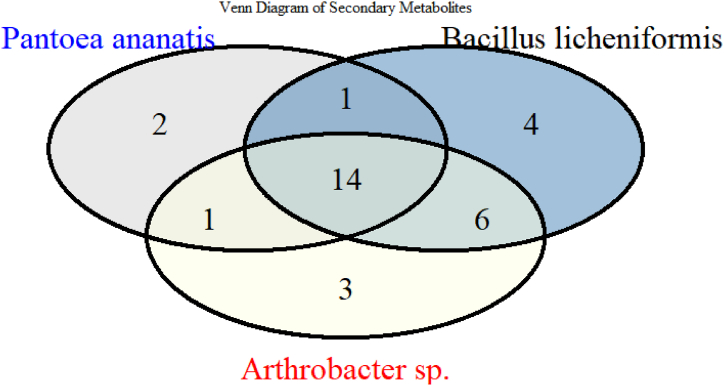
Venn diagram showing the distribution of secondary metabolites among three bacterial endophytes.

The distribution of secondary metabolites from three endophytes, *P. ananatis, B. licheniformis,* and *Arthrobacter* sp., is shown in the Venn diagram below (Fig. 3). The diagram visually represents the overlap and uniqueness of the metabolites produced by each endophyte, highlighting both shared and distinct compounds among the three species. Out of the total metabolites identified, 14 secondary metabolites are common across all three endophytes, indicating a substantial level of metabolic similarity among these bacteria. However, each endophyte also produces unique compounds that are not found in the others, which underscores their distinct biosynthetic capabilities. Specifically, *P. ananatis* and *Arthrobacter* sp., each produce one unique metabolite, while *B. licheniformis* is the most distinct, producing four unique metabolites. Additionally, the diagram reveals the partial overlap between the endophytes. *P. ananatis* and *B. licheniformis* share one metabolite not found in *Arthrobacter* sp., while *Arthrobacter* sp. shares six metabolites with *Bacillus licheniformis* that are not found in *Pantoea ananatis*. Finally, *P. ananatis* and *Arthrobacter* sp. share another metabolite not produced by *B. licheniformis*. This distribution of secondary metabolites not only highlights the shared biosynthetic pathways between the endophytes but also emphasizes their unique metabolic contributions. Such findings are essential in understanding the diversity of bioactive compounds produced by bacterial endophytes and their potential applications in various biotechnological and pharmaceutical fields [2].

Table 1 presents a comprehensive list of secondary metabolites identified from three bacterial endophytes isolated from *S. mauritianum*: *P. ananatis, B licheniformis*, and *Arthrobacter* sp. The table categorizes the metabolites based on their retention time (Rt), mass-to-charge ratio (*m*/*z*), and presence across the three bacterial species. A total of 31 metabolites have been identified, with their occurrence in each endophyte indicated by a "+" sign for presence and a "-" sign for absence (Table 1). The results highlight that Dubinidine, Genipin, Xanthosine, Droserone, 4-Methyl-5-(2′-hydroxyethyl)-thiazole, Anatabine, Khellin, Sinapyl alcohol, Eupaformosanin, Fulvoplumierin, (−)-Laudanidine, Asebogenin, Picropodophyllin, and Protodioscin are universally present in all three endophytes, showcasing a significant overlap in their biosynthetic profiles. This overlap indicates common biosynthetic pathways shared by these endophytes. Conversely, certain metabolites are unique to specific endophytes, underlining the distinct metabolic capabilities of each bacterial species. For instance, 1-Methyluric acid and Sophoraisoflavanone A are uniquely present in *Pantoea ananatis* and *Arthrobacter* sp., respectively, while visnagin, arborinine, rosinidin, vasicinone, mallotophenone, isolobinine, tuliposide A, dauricine, cannabielsoin, cassine, and neoquassin are exclusively found in *Bacillus licheniformis*. The table provides a detailed snapshot of the metabolic diversity and specificity within the endophytic bacteria associated with *S. mauritianum*. The data reflect both the shared and unique secondary metabolites, offering valuable insights into the biosynthetic potential of these microorganisms. This information could be pivotal for further studies aimed at exploring the biotechnological applications of these metabolites, particularly in the development of new pharmaceuticals and agrochemicals. The findings in this study were consistent with the reports of Dat et al. [44], who opined that secondary metabolites from endophytic bacteria can be explored for future drug development, especially those which show great promise with novel compounds.

**Table 1:** Identified secondary metabolites of three selected bacterial endophytes from *S. mauritianum*.

S/N	Rt (min)	(*m*/*z*)	Metabolite name	*P. ananatis*	*B. licheniformis*	*Arthrobacter* sp.
1	2.81	276	Dubinidine	***+***	***+***	***+***
2	2.8	227	Genipin	***+***	***+***	***+***
3	2.89	285	Xanthosine	***+***	***+***	***+***
4	3.15	205	Droserone	***+***	***+***	***+***
5	4.16	144	4-Methyl-5-(2′-hydroxyethyl)-thiazole	***+***	***+***	***+***
6	4.23	161	Anatabine	***+***	***+***	***+***
7	5.74	231	Visnagin	***-***	***+***	***+***
8	5.99	261	Khellin	***+***	***+***	***+***
9	6.58	247	(R)-Columbianetin	***+***	***+***	***+***
10	6.8	183	1-Methyluric acid	***+***	***-***	***+***
11	6.94	211	Sinapyl alcohol	***+***	***+***	***+***
12	6.95	421	Eupaformosanin	***+***	***+***	***+***
13	7.44	245	Fulvoplumierin	***+***	***+***	***+***
14	7.57	371	Sophoraisoflavanone A	***-***	***-***	***+***
15	8.68	286	Arborinine	***-***	***+***	***-***
16	8.78	234	Casimiroin	***-***	***-***	***+***
17	9.16	316	Rosinidin	***-***	***+***	***+***
18	9.31	203	Vasicinone	***-***	***+***	***+***
19	9.33	405	Mallotophenone	***-***	***-***	***+***
20	10.4	344	(−)-Laudanidine	***+***	***+***	***-***
21	11.69	289	Asebogenin	***+***	***+***	***+***
22	16.79	415	Picropodophyllin	***+***	***+***	***+***
23	16.87	273	Naringenin	***+***	***-***	***-***
24	17.29	288	Isolobinine	***-***	***+***	***-***
25	21.48	279	Tuliposide A	***-***	***+***	***+***
26	21.95	625	Dauricine	***-***	***+***	***-***
27	22.2	241	Borrerine	***+***	***-***	***-***
28	25.87	331	Cannabielsoin	***-***	***+***	***+***
29	27.77	298	Cassine	***-***	***+***	***-***
30	29.44	1049	Protodioscin	***+***	***+***	***+***
31	30.62	391	Neoquassin	***-***	***+***	***+***

[M + H]^+^ (*m*/*z*).

**Fig. 4:**
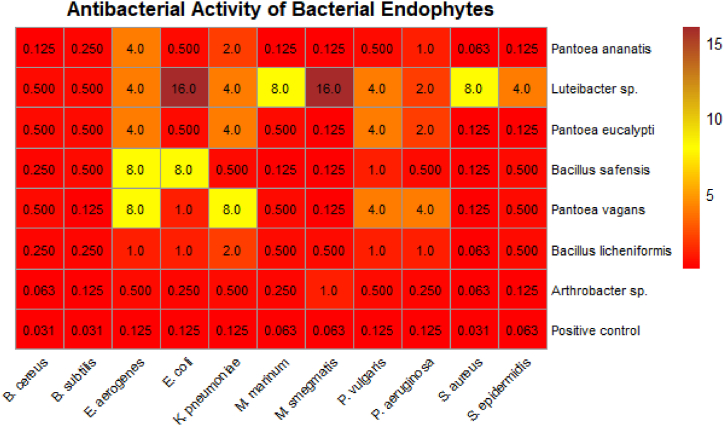
Heatmap of the antibacterial activity/effectiveness (MICs) of crude extracts of bacterial endophytes. The colours of each cell indicate the level of antibacterial activity measured by their respective MICs (in mg/ml except for control (streptomycin) in μg/ml). The colour scale "Red" is indicative of high activity, while "yellow" designates mild (intermediate) or moderate antibacterial activity, and the "brown" colour indicates low antibacterial activity.

The antibacterial activity of crude extracts from the seven isolated bacterial endophytes against multiple public health pathogens is illustrated in the heatmap (Fig. 4). The MICs (measured in mg/ml for all crude endophyte extracts except the control measured in μg/ml) were represented by colour shades with lower MIC values (represented by deep red shading), indicating higher antibacterial activity. The crude extracts of the bacterial endophytes demonstrate broad-spectrum activity efficacy against *Bacillus cereus*, *Pseudomonas aeruginosa*, and *Staphylococcus epidermidis*, where MICs as low as 0.125 mg/ml were recorded across several secondary metabolites (extracts) of *Pantoea ananatis*, *Bacillus licheniformis*, and *Arthrobacter* sp. The efficacy of these endophytes approaches that of the positive control, streptomycin, which exhibits MICs as low as 0.031 mg/ml across all tested pathogens. Notably, certain endophytes such as *Luteibacter* sp. and *Pantoea vagans* display MIC values up to 16 mg/ml, indicating reduced activity against *Escherichia coli* and *Klebsiella pneumoniae*. The gradient of colour intensity across the pathogens emphasizes the variability in endophyte effectiveness, suggesting the potential for specific targeting of bacterial infections based on MIC profiling. This comprehensive MIC analysis positions these bacterial endophytes as promising candidates for further investigation into their role as natural antimicrobials, potentially contributing to the development of novel biocontrol agents in the context of increasing antimicrobial resistance.

**Fig. 5:**
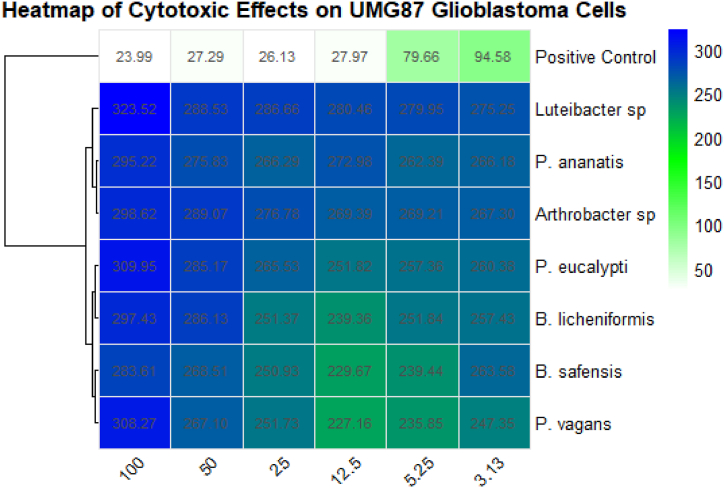
Heatmap showing cell viability (%)/cytotoxic effects of secondary metabolites of bacterial endophytes from *S. mauritianum* on UMG87 glioblastoma cells tested at different concentrations.

This study is among the pioneering investigations on the antibacterial effectiveness of secondary metabolites synthesized by endophytic bacteria found in the aerial portions of *S. mauritianum*. The findings of this study are consistent with the results obtained in the report by Uche-Okereafor et al. [45], who opined that crude secondary metabolites of *Pantoea* species were effective against Gram-positive and negative bacterial isolates such as *E. coli, S. aureus, P. aeruginosa* and *K. pneumoniae.* Also consistent with the results obtained from this study are reports of Akinsanya et al. [46] and Makuwa and Serepa-Dlamini [47], who opined that secondary metabolites from endophytic bacterial isolated from *Aloe vera* and *Docoma anomala,* respectively, were effective against pathogens of public health importance and, as such, are good candidates for use in drug discovery with potential for biotechnological applications. The fact that the crude extract from secondary metabolites of the bacterial endophytes *Pantoea ananatis*, *Bacillus licheniformis*, and *Arthrobacter* sp. showed comparable activity with the control makes them a potential candidate for use in the medical and pharmaceutical industry with further research to explore their mechanism of action and their toxicity to human cells. To further corroborate the results obtained from this study, secondary metabolites of bacterial endophytes were found to be effective against a plethora of pathogens, which have been reported to be of immense significance in medicine, food industry, pharmacology and public health [[48], [49], [50], [51]]. As expected, Ali et al. [52] suggested that secondary metabolites from plant microbiomes might improve environmental sustainability, agricultural output, and antibiotic resistance. A detailed visualization of the cytotoxic effects of secondary metabolites of bacterial endophytes isolated from *Solanum mauritianum* on UMG87 glioblastoma cells is shown in the heatmap (Fig. 5) below. Cell lines were affected by secondary metabolites derived from bacterial endophytes, including *P. ananatis*, Luteibacter sp., *P. eucalypti*, *B. safensis*, *P. vagans*, *B. licheniformis*, and *Arthrobacter* sp., together with auranofin serving as a positive control. The rows of the heatmap represent the bacterial strains and positive control, while the columns represent the concentrations of the secondary metabolites, ranging from 100 μg/mL down to 3.13 μg/mL. Cell viability is represented by the values within the heatmap with the colour gradients used to represent the relative cytotoxicity of the secondary metabolites. The colour scale transitions from white (indicating lower cell viability, meaning higher cytotoxicity) through shades of green and blue and culminates in deep blue (indicating higher cell viability, meaning lower cytotoxicity). The results revealed that all endophytes show some increased cell viability (%) on the UMG87 glioblastoma cells, with the highest concentrations (100 μg/mL) generally resulting in the most pronounced increase in cell viability, as indicated by the deeper blue hues. For instance, *Luteibacter* sp. exhibits the least cytotoxic effects at 100 μg/mL, with cell viability values dropping significantly at lower concentrations but still way higher than the control. Conversely, the positive control (Auranofin) displays markedly lower cell viability across all concentrations, with the most severe effects seen at the highest concentration tested, confirming its potent cytotoxicity relative to the bacterial endophytes. Even at lower concentrations, the positive control continues to show some significant cytotoxic effects, as indicated by the consistently darker shades observed in its corresponding row.

The cytotoxicity assessment of secondary metabolites against UMG87 glioblastoma cells revealed a remarkable difference in cell viability between the metabolites and the control drug, auranofin. At the highest concentration tested (100 μg/mL), the secondary metabolites exhibited an unexpected proliferative effect on the glioblastoma cells, with a cell viability rate of about 300 % on the average of all secondary metabolites. This indicates a threefold increase in cell viability, suggesting that instead of exerting cytotoxic effects, the metabolites may have stimulated cell proliferation under these conditions. Conversely, auranofin, the control drug known for its cytotoxic properties, significantly reduced cell viability to 23.99 %, indicating its expected effectiveness in inducing cell death at this concentration. At the lowest concentration of 3.13 μg/mL, the secondary metabolites still exhibited a high cell viability of about 260 % (on average), while auranofin showed a more moderate reduction in viability at 94.58 %. The consistent high cell viability in the presence of these secondary metabolites across the tested concentrations indicates that these compounds are not cytotoxic to UMG87 glioblastoma cells. The high viability rates also suggest that these metabolites could be involved in pathways that promote cell survival or proliferation rather than inducing cell death, which can pose a significant challenge if they were being considered for anticancer therapy targeting glioblastoma cells. However, this proliferative effect also opens avenues for further research into their potential roles in promoting regeneration or repair in non-cancerous cells, depending on their mechanism of action.

**Fig. 6:**
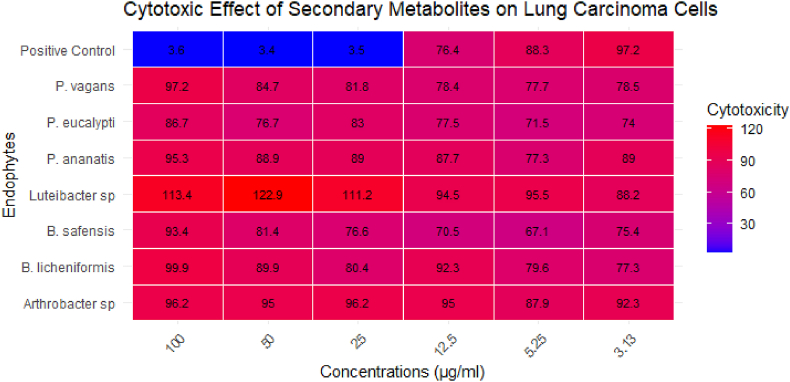
Heatmap showing cytotoxic effects of secondary metabolites of bacterial endophytes from *S. mauritianum* on A549 lung carcinoma cells tested at different concentrations.

The effects of the secondary metabolites produced by the endophytes were also tested on the A549 lung carcinoma cell line, and the result is shown in the heatmap (Fig. 6) below. The colour gradient from blue to red indicates the level of cytotoxicity, with red representing lower cytotoxic effects and blue indicating higher cytotoxicity (by their lower cell viability values). The endophytes exhibit diverse responses, with some, like *Luteibacter* sp**.**, showing significantly higher cell viability values at higher concentrations (indicated by the more intense red colouring). In contrast, other endophytes, such as *Arthrobacter* sp. and *B. licheniformis,* demonstrated moderate to lower values for cell viability across the tested concentrations. The positive control, however, displayed a high cytotoxic effect across most concentrations.

The results of the same secondary metabolites on A549 lung carcinoma cells provided contrasting results when compared to the UMG87 glioblastoma cells, revealing a significantly lower cytotoxic effect. At the highest concentration of 100 μg/mL, cell viability was 95 % in the presence of the metabolites, indicating a minimal cytotoxic effect. This result starkly contrasts with the control, which demonstrated potent cytotoxicity with only 3.6 % cell viability, confirming the effectiveness of the control drug in killing lung carcinoma cells. At the lowest concentration of 3.13 μg/mL, the metabolites showed a slight reduction in cell viability to 92 %, which is still very close to the viability observed with the control at this concentration, which was 95 %. The relatively high cell viability across both concentrations suggests that these secondary metabolites lack significant cytotoxic activity against A549 lung carcinoma cells, similar to the observations in glioblastoma cells. This low cytotoxicity indicates that these metabolites may not be suitable for direct application in anticancer drug development aimed at lung carcinoma, as they do not effectively induce cell death in these cancer cells. However, the low cytotoxicity and high viability at various concentrations suggest that these metabolites might have potential in other therapeutic areas where preserving cell viability is crucial, or they could be further modified to enhance their cytotoxic effects against cancer cells. Future studies should explore the specific pathways and mechanisms these metabolites influence, which could inform their potential utility in drug discovery, possibly as adjuvants or in combination therapies where reducing the toxic side effects of other agents is desirable.

The results obtained for the cytotoxicity of crude secondary metabolites from bacterial endophytes against glioblastoma and lung carcinoma cells were at variance with some of the reports in literature for certain metabolites produced by bacteria endophytes with potent cytotoxic effects especially against lung carcinoma cells [[53], [54], [55], [56]]. Interestingly, all of the reports in the studies mentioned above were essentially endophytes (actinomycetes) isolated from other plants and marine environments. More so, the reports by Tapfuma et al. [20] for one of its secondary metabolites were found to have significant cytotoxicity against both glioblastoma and lung carcinoma cell lines. However, the secondary metabolites were produced by fungi in contrast to the metabolites evaluated in this study. There are also reports which support the findings of the results obtained in this study, as the high cell viability (low cytotoxicity) observed by the crude bacterial endophytes was also what was obtained in their report [45,57].

## 4 Conclusion

The study provides valuable insights into the diversity and tissue-specific distribution of bacterial endophytes associated with *S. mauritianum* Scop. The presence of endophytes from three distinct bacterial classes highlights the influence of plant tissue type on endophytic communities. The identification of secondary metabolites with both shared and unique biosynthetic pathways among the endophytes emphasizes their potential for novel bioactive compound discovery. The antibacterial activity of these metabolites demonstrated broad-spectrum efficacy against significant public health pathogens, positioning these endophytes as promising candidates for antimicrobial development. However, the cytotoxicity assessment against UMG87 glioblastoma cells unveiled an unexpected proliferative effect rather than the desired cytotoxic outcome, indicating the need for further investigation into the underlying mechanisms of these secondary metabolites. While their non-cytotoxic nature may limit their direct application as anticancer agents, it also opens up new research avenues for their potential use in promoting cellular regeneration or repair in non-cancerous contexts. Generally, the findings contribute to the understanding of plant-microbe interactions and underscore the potential of endophytic bacteria as sources of novel bioactive compounds with diverse applications in biotechnology and medicine.

## CRediT authorship contribution statement

**Abraham Goodness Ogofure:** Writing – review & editing, Writing – original draft, Visualization, Data curation. **Ezekiel Green:** Validation, Supervision, Methodology, Conceptualization.

## Data and code availability

The data that support the findings of this study will be made available by the corresponding author upon reasonable request.

## Funding

This research was funded by the University Research Committee of the University of Johannesburg, Project No. 075432.

## Declaration of competing interest

The authors declare that they have no known competing financial interests or personal relationships that could have appeared to influence the work reported in this paper.
